# Can we turn off the “Covid-biting-tail” inflammation?

**DOI:** 10.37825/2239-9747.1006

**Published:** 2020-10-01

**Authors:** Giudice Valentina, Filippelli Amelia, Selleri Carmine

**Affiliations:** 1 Department of Medicine, Surgery and Dentistry “Scuola Medica Salernitana”, University of Salerno, Italy; 2 Clinical Pharmacology, University Hospital “San Giovanni di Dio e Ruggi d’Aragona”, Italy; 3 Hematology and Transplant Center, University Hospital “San Giovanni di Dio e Ruggi d’Aragona”, Italy

**Keywords:** SARS-CoV-2, Covid-19, inflammation

Dear Editor,

More than 30% of subjects with Covid-19 requires intensive care unit admission, and in several cases, patients develop severe respiratory symptoms and an acute respiratory distress syndrome (ARDS) requiring intensive care treatment, and precipitating factors are still under investigation; however, a massive release of pro-inflammatory cytokines and chemokines from immune cells is one of the main pathogenetic mechanisms.1 Based on previous findings in SARS-CoV and Middle East respiratory syndrome CoV diseases, infected macrophages can belatedly release high amount of pro-inflammatory cytokines, especially TNFα and IL-6, and sustain and enhance inflammatory and immune responses during SARS-CoV-2 infection contributing to ARDS development and subsequently to the multiorgan failure syndrome (MOFS) [1].

The main viral entry mechanism is the binding of the viral “Spike” protein to the surface Angiotensin-Converting Enzyme 2 (ACE-2) receptor on host alveolar epithelial cells. ACE-2, a zinc containing type I transmembrane metalloenzyme, regulates blood pressure through the renin-angiotensin system (RAS), and is highly expressed on various cell types, such as vascular endothelial cells and alveolar epithelial cells [2]. Therefore, SARS-CoV-2 virus infects alveolar cells through ACE-2 receptor binding sequestrating available receptors for RAS regulation and increasing kinin, complement, and coagulation cascade activation. The complement system, a multi-step pathway of the innate immune response, causes the formation of pores on pathogen membranes inducing lysis and enhancing phagocytosis by macrophages, and releases several pro-inflammatory molecules, the anaphylatoxins, that amplify inflammation and immune responses [1–2]. Some of those molecules can trigger the activation of kinin and coagulation cascades which can sustain vascular permeability and thrombosis also occurring during ARDS. In addition, vascular permeability facilitates neutrophil and macrophage recruitment at the site of viral infection supporting inflammation and tissue damage. Simultaneously, infected cells trigger adaptive immune responses, such as Th1 with release of type I interferons, macrophage recruitment and activation with release of IL-1 and TNFα. These mechanisms might increase vascular permeability and neutrophil recruitment in the site of viral infection with production of large amount of pro-inflammatory cytokines, especially IL-6, and anaphylatoxins which sustain inflammation and tissue damage as in a “dog-biting-tail” system until pulmonary edema, thrombosis and respiratory distress develop and progress to ARDS and MOFS (Figure 1).

Currently, there are no specific therapies for Covid-19; however, a lot of available drugs used in other diseases, especially autoimmune disorders, are under investigation for treatment of severe SARS-CoV-2 infection or for prophylaxis [3]. In particular, eculizumab, an anti-C5a complement factor monoclonal antibody used for treatment of paroxysmal nocturnal hemoglobinuria, is currently under investigation in the United States (SOLID-C19 trial NCT04288713) and Italy for treatment of Covid-19. Growing evidence of efficacy of eculizumab in SARS-CoV-2-related ARDS are accumulating showing a potential benefit of this drug in reducing platelet consumption and intravascular coagulation thus preventing thrombosis of pulmonary capillaries and interstitial pneumonia or reducing ongoing pulmonary lesions [4]. In addition, Janus kinase (JAK) inhibitors are under investigation because of the wide involvement of JAK/STAT pathway in transducing the signals of several cytokine stimulation, such as interferons and TNFα [3]. In 2019, the Food and Drug Administration has approved ruxolitinib, a JAK1/2 inhibitor, for treatment of Graft-versus-Host disease (GvHD) because ruxolitinib has shown *in vitro* and *in vivo* efficacy in modulating immune responses, especially those exerted by Natural Killer cells, DCs, Th1 and Th17 cells, and regulatory T lymphocytes, and in regulating release of several cytokines including TNFα. Moreover, ruxolitinib can reduce viral replication, as already described for HIV in in vitro and mouse models. Preliminary results have described a fast clinical recovery of Covid-19 patients treated with ruxolitinib compared to placebo; however, JAK inhibition might cause severe adverse events, including cutaneous rash.

Based on the hypothesis that SARS-CoV-2 infection triggers a “dog-biting-tail” inflammatory loop, the use of a single anti-inflammatory agent might be not sufficient in severe Covid-19; thus, a combination of drugs would be advisable in preventing ARDS and MOFS in intensive care patients. For example, we have described clinical improvements of six severe Covid-19 cases treated with ruxolitinib 10 mg/twice daily for 14 days and eculizumab 900 mg IV/weekly for three weeks [5].

Patients treated with the combination showed significant improvements in respiratory symptoms and radiographic pulmonary lesions, and decreased levels of circulating D-dimers while significant increase in platelet counts. Therefore, eculizumab could reduce complement and coagulation cascade activation and decrease vascular permeability and thrombosis on one side; while ruxolitinib might inhibit Th1 and macrophage activation ultimately leading to reduced neutrophil recruitment in the site of viral infection. In conclusion, combined use of anti-inflammatory drugs acting on different but connected pathways during severe Covid-19 might dramatically turn off inflammation in the lungs thus reducing the risk of ARDS and MOFS development.

## Figures and Tables

**Fig. 1:**
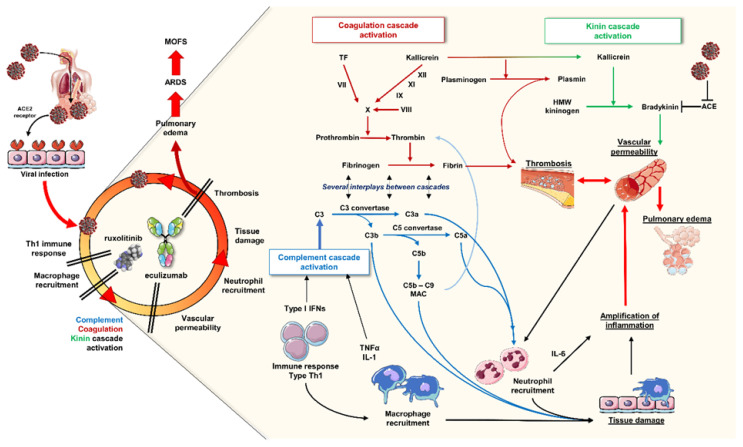
The “Covid-biting-tail” inflammation. SARS-CoV-2 infects alveolar cells by binding ACE-2 receptor eliciting a T helper 1 immune response and activation of macrophages with release of type I interferons (IFNs), interleukin(IL)-1, and tumor necrosis factor alpha (TNFα), contributing to complement cascade activation and tissue damage. Several products, such as the membrane attack complex (MAC), can enhance the activation of prothrombin to thrombin leading to thrombotic events, and can sustain tissue damage by facilitating macrophage phagocytosis. These events might trigger (or perpetuate) coagulation cascade which also sustains complement system activation. C3a and C5a, also known as anaphylatoxins, contribute together with macrophages and Th1 cells to neutrophil recruitment in the site of viral infection and amplification of inflammatory responses by releasing of several pro-inflammatory mediators. However, increased vascular permeability is required to facilitate neutrophil recruitment: kinin cascade products play an important role in regulating vascular permeability, and ACE enzymes can degrade those molecules in non-active peptides. Therefore, viral occupancy of ACE-2 receptors might reduce regulatory ACE functions, and increase vascular permeability leading to pulmonary edema. Moreover, increased vascular permeability favors thrombosis and neutrophil recruitment ultimately leading to persistence of inflammation and tissue damage. Ruxolitinib, a JAK1/2 inhibitor, and eculizumab, an anti-C5a monoclonal antibody, can block this inflammatory circle in multiple steps (some artworks from https://smart.servier.com).
